# Supplementary material for: Hydrogen sulfide exposure reduces thermal set point in zebrafish
Source: R Soc Open Sci. 2020 Nov 4;7(11):200416. doi: 10.1098/rsos.200416 (PMC7735326; doi:10.1098/rsos.200416)
Supplement: Statistical Model Summaries [file rsos200416supp1.pdf]

Model summaries for Skandalis et al.

Dimitri A Skandalis

03 June 2020

Contents

1 ANALYSIS OF CONSTANT TEMPERATURE **3**

1.1 SHUTTLING AT CONSTANT TEMPERATURE . . . 3

1.1.1 Generalised additive model of shuttles ~ Time . . . 3

2 ANALYSIS OF H_2S **5**

2.1 POPULATION-AVERAGE BODY TEMPERATURE OVER TIME AND H_2S . . . 5

2.1.1 Generalised additive model of population fish temperature ~ Time (hrs) x H_2S . . . 6

2.2 AVERAGE TREATMENT EFFECTS . . . 6

2.2.1 Fish temperature ~ H_2S . . . 6

2.2.2 Lower escape temperature ~ H_2S . . . 8

2.2.3 Upper escape temperature ~ H_2S . . . 8

2.2.4 log Shuttle rate ~ H_2S . . . 9

2.2.5 log Swim velocity ~ H_2S . . . 9

2.2.6 Time ratio ~ H_2S . . . 10

2.3 REPEATABILITY (0% H_2S) . . . 10

2.3.1 Fish temperature ramping->testing repeatability . . . 10

2.3.2 Fish log shuttle rate ramping->testing repeatability . . . 11

2.4 RESPONSES IN RAMPING AND TESTING PHASES . . . 11

2.4.1 Fish temperature (testing) ~ Fish temperature (ramping) x H_2S level . . . 11

2.4.2 log Shuttle rate (testing) ~ log Shuttle rate (ramping) x H_2S level . . . 12

2.4.3 Lower escape temperature (testing) ~ Lower escape temperature (ramping) x H_2S level 12

2.4.4 Upper escape temperature (testing) ~ Upper escape temperature (ramping) x H_2S level 13

2.4.5 Swim velocity (testing) ~ Swim velocity (ramping) x H_2S level . . . 13

2.5 AQUATIC SURFACE RESPIRATION RATES BY TEMPERATURE AND H_2S LEVEL . . . 14

2.5.1 Generalised linear model of ASR ~ Temperature x H_2S . . . 14

2.5.2 Fish temperature ~ log Shuttle rate x H_2S level . . . 14

2.5.3 Fish temperature ~ Swim velocity x H_2S level . . . 15

2.5.4 Fish temperature ~ Time ratio x H_2S level . . . 15

3 REANALYSIS OF H_2S (Short time series) 17

3.1 AVERAGE TREATMENT EFFECTS 17

3.1.1 Fish temperature ~ H_2S 17

3.1.2 Lower escape temperature ~ H_2S 17

3.1.3 Upper escape temperature ~ H_2S 18

3.1.4 log Shuttle rate ~ H_2S 18

3.1.5 log Swim velocity ~ H_2S 19

3.1.6 Time ratio ~ H_2S 19

3.2 REPEATABILITY (0% H_2S) 20

3.2.1 Fish temperature ramping->testing repeatability 20

3.2.2 Fish log shuttle rate ramping->testing repeatability 20

3.3 RESPONSES IN RAMPING AND TESTING PHASES 21

3.3.1 Fish temperature (testing) ~ Fish temperature (ramping) x H_2S level 21

3.3.2 log Shuttle rate (testing) ~ log Shuttle rate (ramping) x H_2S level 21

3.3.3 Lower escape temperature (testing) ~ Lower escape temperature (ramping) x H_2S level 22

3.3.4 Upper escape temperature (testing) ~ Upper escape temperature (ramping) x H_2S level 22

3.3.5 Swim velocity (testing) ~ Swim velocity (ramping) x H_2S level 23

4 ANALYSIS OF HYPOXIA (2% O_2) 24

4.1 AVERAGE TREATMENT EFFECTS 24

4.1.1 Fish temperature ~ O_2 24

4.1.2 Lower escape temperature ~ O_2 24

4.1.3 Upper escape temperature ~ O_2 24

4.1.4 Swim velocity ~ O_2 25

4.1.5 log Shuttle rate ~ O_2 25

4.1.6 Side preference ~ O_2 25

1 ANALYSIS OF CONSTANT TEMPERATURE

Shuttlebox data collected by Joshua C. Shaw

Analysis performed by Dimitri A. Skandalis

1.1 SHUTTLING AT CONSTANT TEMPERATURE

We counted the number of shuttles in 1 min intervals, allowing a nearly continuous measure of behaviour over the course of an experiment. This measurement of behaviour was selected to reflect the habituation timeline of fish to the shuttlebox alone, in the absence of any other effects.

1.1.1 Generalised additive model of shuttles ~ Time

```
##
## Family: Zero inflated Poisson(-1.975,2.198)
## Link function: identity
##
## Formula:
## Shuttles ~ s(minute, bs = "gp") + s(minute, fishID, bs = "fs",
##     m = 1)
##
## Parametric coefficients:
##             Estimate Std. Error t value Pr(>|t|)
## (Intercept)   1.0414     0.1761   5.913 3.67e-09 ***
## ---
## Signif. codes:  0 '***' 0.001 '**' 0.01 '*' 0.05 '.' 0.1 ' ' 1
##
## Approximate significance of smooth terms:
##                     edf Ref.df      F p-value
## s(minute)         5.991   6.67  1.955  0.0999 .
## s(minute,fishID) 46.854  71.00 20.833  <2e-16 ***
## ---
## Signif. codes:  0 '***' 0.001 '**' 0.01 '*' 0.05 '.' 0.1 ' ' 1
##
## Deviance explained = 41.2%
## fREML =  10479  Scale est. = 1         n = 3823
```

Autocorrelation ρ manually selected to minimise autocorrelation of the residuals.

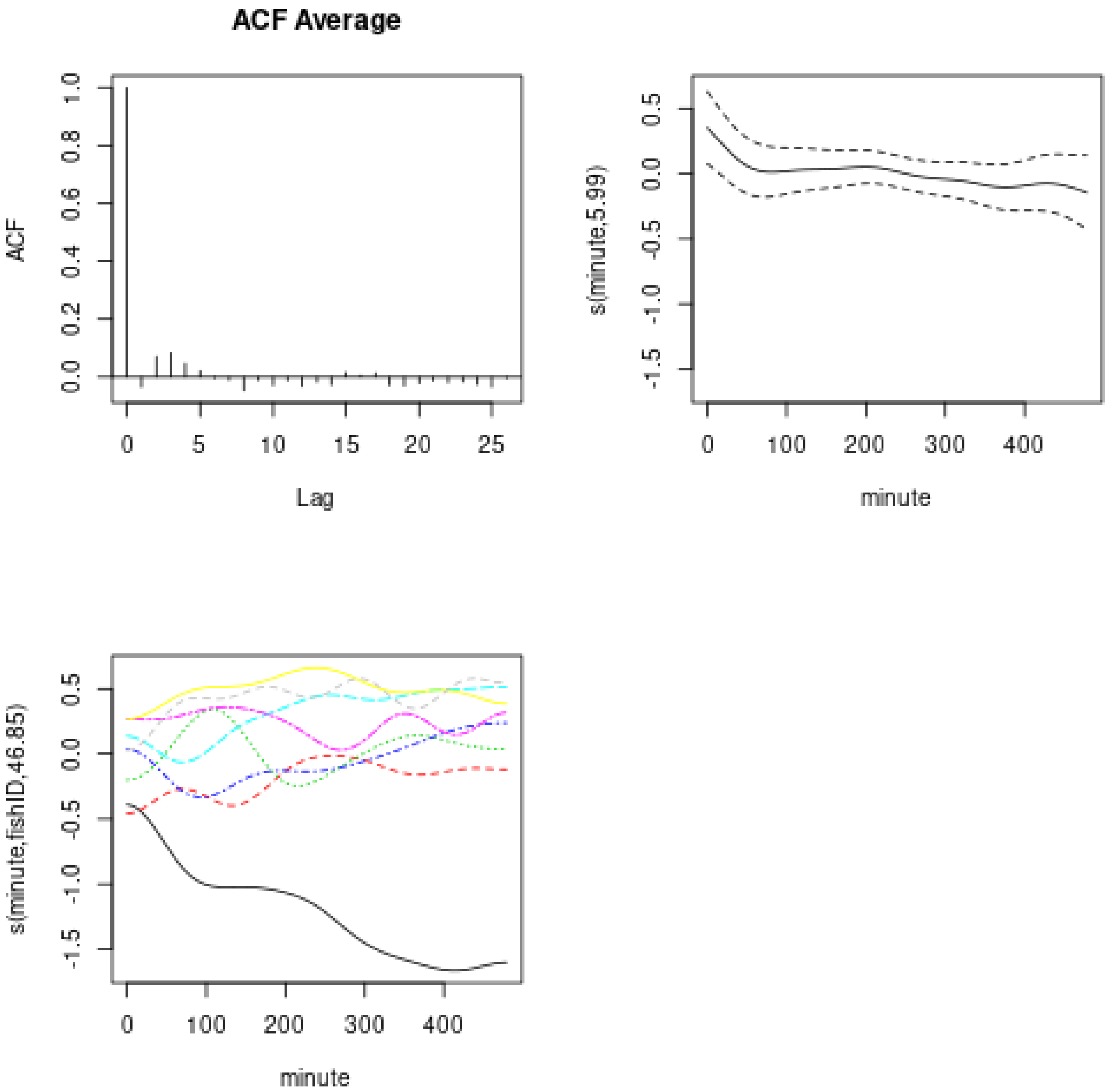

Figure 1: Model residual autocorrelations and fits

2 ANALYSIS OF H_2S

Aquatic surface respiration data collected by Glenn J. Tattersall

Shuttlebox data collected by Cheryl D. Dobell

Analysis performed by Dimitri A. Skandalis

2.1 POPULATION-AVERAGE BODY TEMPERATURE OVER TIME AND H_2S

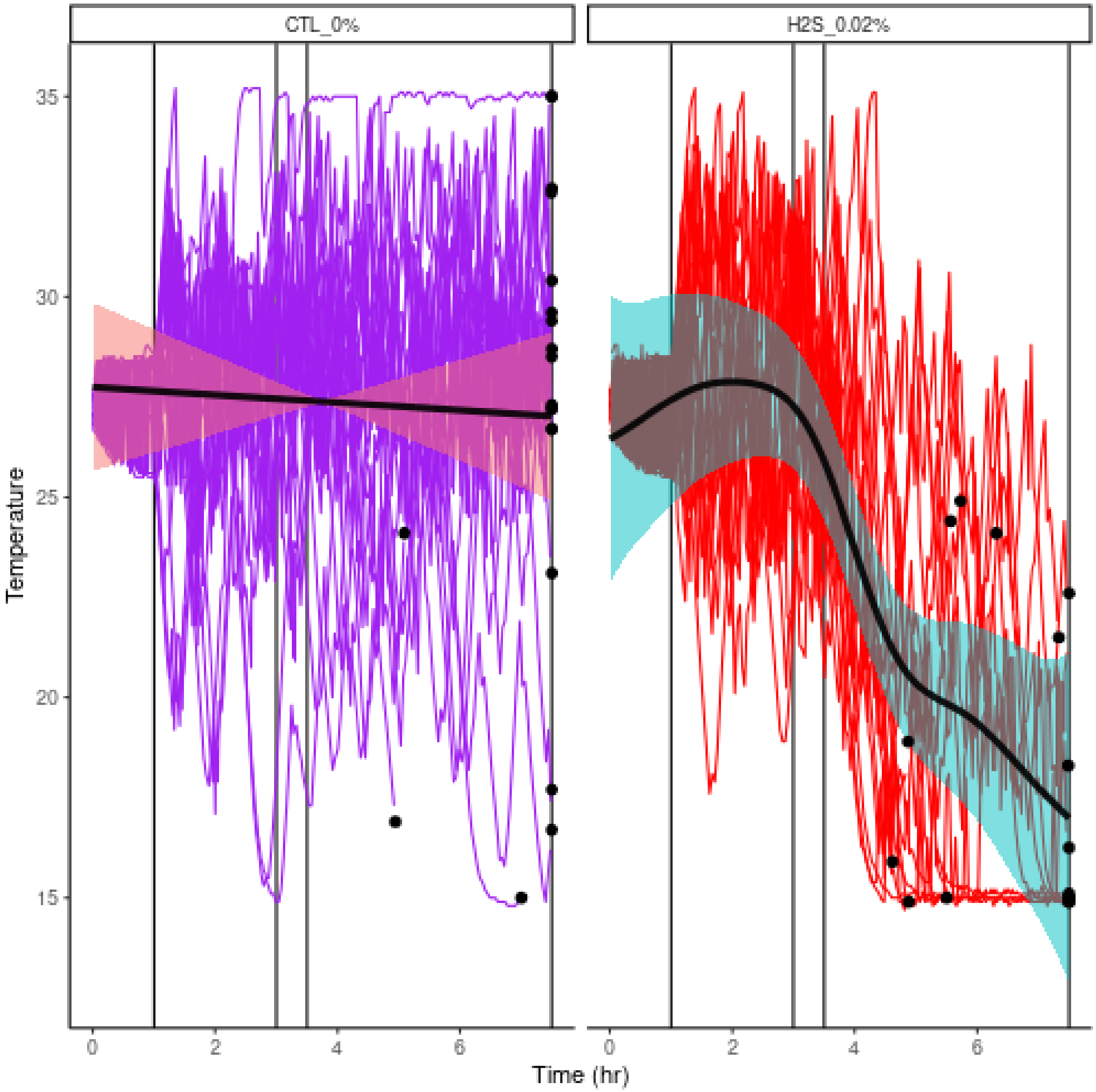

Figure 2: Body temperature of fish in control and H_2S conditions. Black points denote stopping times for individual fish; points may be disconnected from individual traces due to subsampling (1/50) of the traces.

2.1.1 Generalised additive model of population fish temperature ~ Time (hrs) x H_2S

```
##
## Family: gaussian
## Link function: identity
##
## Formula:
## Temp.fish ~ s(t.hr, bs = "gp") + s(t.hr, by = Treat_level, bs = "gp") +
##     s(t.hr, fishID, bs = "fs") + Treat_level
##
## Parametric coefficients:
##               Estimate Std. Error t value Pr(>|t|)
## (Intercept)    25.4897     0.4344  58.675  < 2e-16 ***
## Treat_level.L  -2.6773     0.6144  -4.358 1.31e-05 ***
## ---
## Signif. codes:  0 '***' 0.001 '**' 0.01 '*' 0.05 '.' 0.1 ' ' 1
##
## Approximate significance of smooth terms:
##                                edf  Ref.df      F p-value
## s(t.hr)                      1.009   1.016  0.452   0.501
## s(t.hr):Treat_levelH2S_0.02%  6.234   7.451 16.494  <2e-16 ***
## s(t.hr,fishID)              69.698 368.000  1.417  <2e-16 ***
## ---
## Signif. codes:  0 '***' 0.001 '**' 0.01 '*' 0.05 '.' 0.1 ' ' 1
##
## R-sq.(adj) =  0.698   Deviance explained = 69.8%
## fREML = 4.092e+05  Scale est. = 23.946    n = 390179
```

Autocorrelation ρ manually selected to minimise autocorrelation of the residuals.

2.2 AVERAGE TREATMENT EFFECTS

2.2.1 Fish temperature ~ H_2S

```
##
## Call:
## lm(formula = Temp.fish ~ Treat_level, data = dat_CDD_testphase)
##
## Residuals:
##     Min      1Q  Median      3Q     Max
## -6.6897 -2.5802  0.2867  1.8745  7.4603
##
## Coefficients:
##                      Estimate Std. Error t value Pr(>|t|)
## (Intercept)           27.2977     0.7916  34.485  < 2e-16 ***
## Treat_levelH2S_0.02%  -6.0016     1.1678  -5.139 1.05e-05 ***
## ---
## Signif. codes:  0 '***' 0.001 '**' 0.01 '*' 0.05 '.' 0.1 ' ' 1
##
## Residual standard error: 3.54 on 35 degrees of freedom
## Multiple R-squared:  0.4301, Adjusted R-squared:  0.4138
## F-statistic: 26.41 on 1 and 35 DF,  p-value: 1.053e-05
```

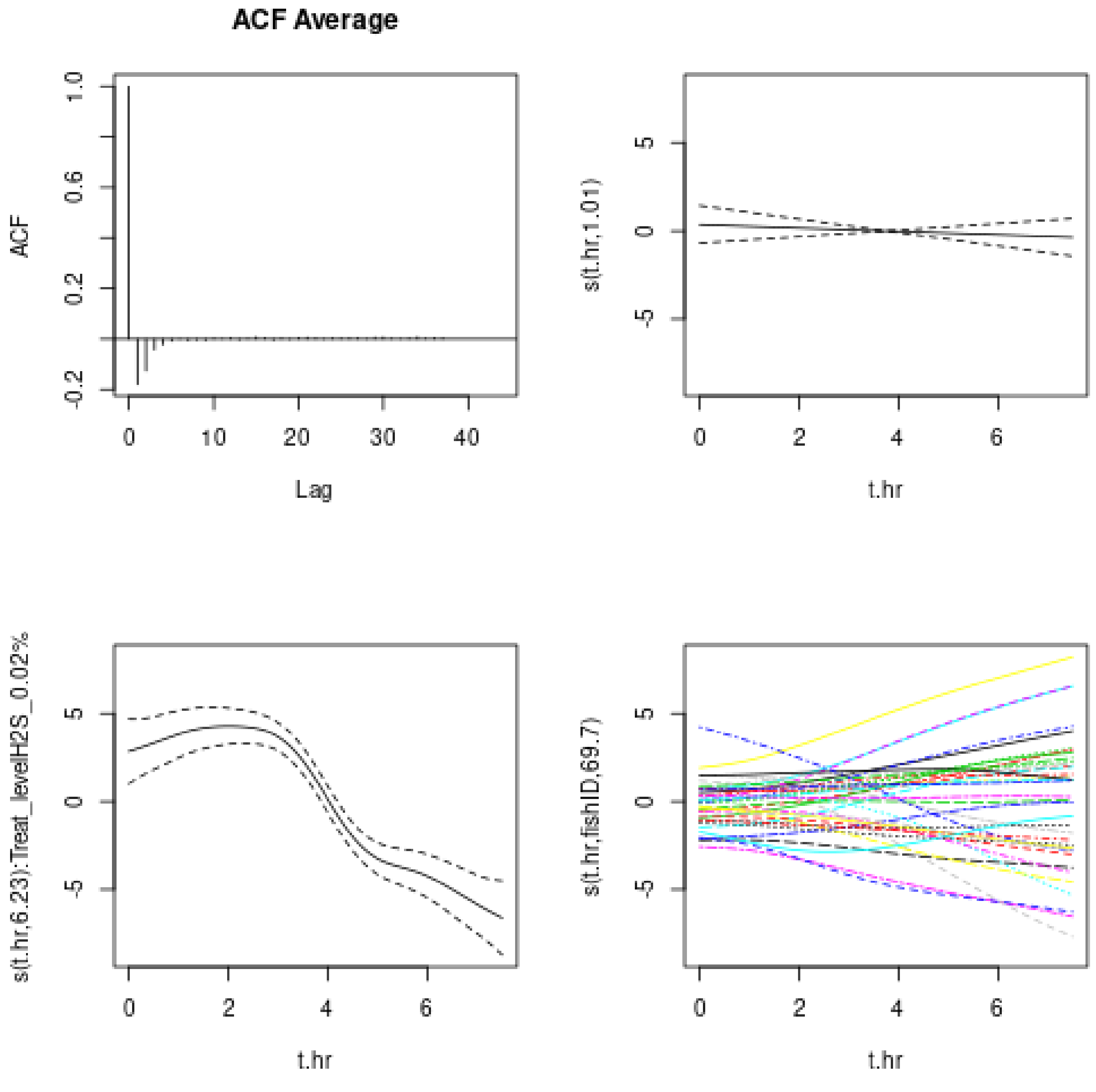

Figure 3: Model residual autocorrelations and fits

Confidence intervals:

```
##                          2.5 %   97.5 %
## (Intercept)          25.690658 28.90468
## Treat_levelH2S_0.02% -8.372442 -3.63084
```

2.2.2 Lower escape temperature ~ H_2S

```
##
## Call:
## lm(formula = LET ~ Treat_level, data = dat_CDD_testphase)
##
## Residuals:
##     Min      1Q  Median      3Q     Max
## -7.2700 -2.4587  0.8313  2.2224  6.1822
##
## Coefficients:
##                      Estimate Std. Error t value Pr(>|t|)
## (Intercept)           25.9272     0.7545  34.361  < 2e-16 ***
## Treat_levelH2S_0.02%  -3.8111     1.1132  -3.424  0.00159 **
## ---
## Signif. codes:  0 '***' 0.001 '**' 0.01 '*' 0.05 '.' 0.1 ' ' 1
##
## Residual standard error: 3.374 on 35 degrees of freedom
## Multiple R-squared:  0.2509, Adjusted R-squared:  0.2295
## F-statistic: 11.72 on 1 and 35 DF,  p-value: 0.001591
```

Confidence intervals:

```
##                          2.5 %    97.5 %
## (Intercept)          24.395354 27.458972
## Treat_levelH2S_0.02% -6.070921 -1.551207
```

2.2.3 Upper escape temperature ~ H_2S

```
##
## Call:
## lm(formula = UET ~ Treat_level, data = dat_CDD_testphase)
##
## Residuals:
##     Min      1Q  Median      3Q     Max
## -8.3378 -2.3257  0.7727  2.1422  6.4464
##
## Coefficients:
##                      Estimate Std. Error t value Pr(>|t|)
## (Intercept)           29.3092     0.8228  35.622  < 2e-16 ***
## Treat_levelH2S_0.02%  -4.2056     1.2138  -3.465  0.00142 **
## ---
## Signif. codes:  0 '***' 0.001 '**' 0.01 '*' 0.05 '.' 0.1 ' ' 1
##
## Residual standard error: 3.68 on 35 degrees of freedom
## Multiple R-squared:  0.2554, Adjusted R-squared:  0.2341
## F-statistic:    12 on 1 and 35 DF,  p-value: 0.001421
```

Confidence intervals:

```
##                            2.5 %    97.5 %
## (Intercept)          27.638914 30.979563
## Treat_levelH2S_0.02% -6.669834 -1.741419
```

2.2.4 log Shuttle rate ~ H_2S

```
##
## Call:
## lm(formula = log_Shuttle_rt ~ Treat_level, data = dat_CDD_testphase)
##
## Residuals:
##      Min       1Q   Median       3Q      Max
## -0.89466 -0.36173 -0.00825  0.32151  0.84276
##
## Coefficients:
##                      Estimate Std. Error t value Pr(>|t|)
## (Intercept)           1.62893    0.10541  15.454   <2e-16 ***
## Treat_levelH2S_0.02%  0.08226    0.15550   0.529      0.6
## ---
## Signif. codes:  0 '***' 0.001 '**' 0.01 '*' 0.05 '.' 0.1 ' ' 1
##
## Residual standard error: 0.4714 on 35 degrees of freedom
## Multiple R-squared:  0.007931,   Adjusted R-squared:  -0.02041
## F-statistic: 0.2798 on 1 and 35 DF,  p-value: 0.6002
```

Confidence intervals:

```
##                           2.5 %   97.5 %
## (Intercept)           1.4149457 1.842918
## Treat_levelH2S_0.02% -0.2334347 0.397947
```

2.2.5 log Swim velocity ~ H_2S

```
##
## Call:
## lm(formula = log(Velocity) ~ Treat_level, data = dat_CDD_testphase)
##
## Residuals:
##     Min      1Q  Median      3Q     Max
## -0.8365 -0.2284 -0.1433  0.2824  1.4661
##
## Coefficients:
##                      Estimate Std. Error t value Pr(>|t|)
## (Intercept)           -1.4661     0.1167  -12.57 1.56e-14 ***
## Treat_levelH2S_0.02%   0.4905     0.1721    2.85  0.00727 **
## ---
## Signif. codes:  0 '***' 0.001 '**' 0.01 '*' 0.05 '.' 0.1 ' ' 1
##
## Residual standard error: 0.5217 on 35 degrees of freedom
## Multiple R-squared:  0.1884, Adjusted R-squared:  0.1652
## F-statistic: 8.124 on 1 and 35 DF,  p-value: 0.007274
```

Confidence intervals (on metric scale):

```
##     2.5 %    97.5 %
## 0.2097651 0.6774714
```

2.2.6 Time ratio ~ H_2S

```
##
## Call:
## lm(formula = Time_ratio ~ Treat_level - 1, data = dat_CDD_testphase)
##
## Residuals:
##      Min       1Q   Median       3Q      Max
## -0.55589 -0.10930  0.00114  0.13199  0.84458
##
## Coefficients:
##                      Estimate Std. Error t value Pr(>|t|)
## Treat_levelCTL_0%     0.05667    0.06216   0.912    0.368
## Treat_levelH2S_0.02% -0.34491    0.06742  -5.116 1.13e-05 ***
## ---
## Signif. codes:  0 '***' 0.001 '**' 0.01 '*' 0.05 '.' 0.1 ' ' 1
##
## Residual standard error: 0.278 on 35 degrees of freedom
## Multiple R-squared:  0.4355, Adjusted R-squared:  0.4033
## F-statistic:  13.5 on 2 and 35 DF,  p-value: 4.508e-05
```

Confidence intervals:

```
##                            2.5 %     97.5 %
## Treat_levelCTL_0%    -0.06952303  0.1828530
## Treat_levelH2S_0.02% -0.48178175 -0.2080415
```

2.3 REPEATABILITY (0% H_2S)

2.3.1 Fish temperature ramping->testing repeatability

```
##
## Repeatability estimation using the lmm method
##
## Call = rptR::rptGaussian(formula = Temp.fish ~ (1 | fishID), grname = "fishID", data = dat_icc, paral
##
## Data: 40 observations
## ----------------------------------------
##
## fishID (20 groups)
##
## Repeatability estimation overview:
##       R     SE   2.5%  97.5% P_permut  LRT_P
##   0.539  0.161  0.139  0.783       NA  0.006
##
## Bootstrapping and Permutation test:
##             N   Mean Median   2.5%  97.5%
```

```
## boot     1000  0.522  0.541  0.139  0.783
## permut      1     NA     NA     NA     NA
##
## Likelihood ratio test:
## logLik full model = -96.16956
## logLik red. model = -99.33616
## D  = 6.33, df = 1, P = 0.00592
##
## ----------------------------------------
```

2.3.2 Fish log shuttle rate ramping->testing repeatability

```
##
## Repeatability estimation using the lmm method
##
## Call = rptR::rptGaussian(formula = log_Shuttle_rt ~ (1 | fishID), grname = "fishID", data = dat_icc,
##
## Data: 40 observations
## ----------------------------------------
##
## fishID (20 groups)
##
## Repeatability estimation overview:
##       R     SE   2.5%  97.5% P_permut  LRT_P
##   0.508  0.172 0.0779  0.756       NA   0.01
##
## Bootstrapping and Permutation test:
##             N   Mean Median   2.5%  97.5%
## boot     1000  0.487   0.51 0.0779  0.756
## permut      1     NA     NA     NA     NA
##
## Likelihood ratio test:
## logLik full model = -32.38444
## logLik red. model = -35.11355
## D  = 5.46, df = 1, P = 0.00974
##
## ----------------------------------------
```

2.4 RESPONSES IN RAMPING AND TESTING PHASES

2.4.1 Fish temperature (testing) ~ Fish temperature (ramping) x H_2S level

```
##
## Call:
## lm(formula = Temp.fish_Test ~ Temp.fish_Ramping * Treat_level,
##     data = dat_period)
##
## Residuals:
##     Min      1Q  Median      3Q     Max
## -7.4145 -2.6392 -0.1962  1.7412  7.2704
##
## Coefficients:
##                                       Estimate Std. Error t value Pr(>|t|)
```

```
## (Intercept)                               2.8459     0.7033   4.047 0.000295 ***
## Temp.fish_Ramping                         0.8832     0.3155   2.799 0.008488 **
## Treat_levelH2S_0.02%                     -6.1661     1.0377  -5.942 1.14e-06 ***
## Temp.fish_Ramping:Treat_levelH2S_0.02%  -0.2362     0.4646  -0.508 0.614562
## ---
## Signif. codes:  0 '***' 0.001 '**' 0.01 '*' 0.05 '.' 0.1 ' ' 1
##
## Residual standard error: 3.142 on 33 degrees of freedom
## Multiple R-squared:  0.5767, Adjusted R-squared:  0.5383
## F-statistic: 14.99 on 3 and 33 DF,  p-value: 2.508e-06
```

2.4.2 log Shuttle rate (testing) ~ log Shuttle rate (ramping) x H_2S level

```
##
## Call:
## lm(formula = log_Shuttle_rt_Test ~ log_Shuttle_rt_Ramping * Treat_level,
##     data = dat_period)
##
## Residuals:
##      Min       1Q   Median       3Q      Max
## -0.86440 -0.27410  0.03254  0.27154  0.70360
##
## Coefficients:
##                                              Estimate Std. Error t value Pr(>|t|)
## (Intercept)                                   0.02243    0.08278   0.271    0.788
## log_Shuttle_rt_Ramping                        0.67825    0.14773   4.591 6.12e-05 ***
## Treat_levelH2S_0.02%                         -0.02381    0.12336  -0.193    0.848
## log_Shuttle_rt_Ramping:Treat_levelH2S_0.02% -0.23932    0.26150  -0.915    0.367
## ---
## Signif. codes:  0 '***' 0.001 '**' 0.01 '*' 0.05 '.' 0.1 ' ' 1
##
## Residual standard error: 0.3655 on 33 degrees of freedom
## Multiple R-squared:  0.4376, Adjusted R-squared:  0.3865
## F-statistic: 8.561 on 3 and 33 DF,  p-value: 0.0002409
```

2.4.3 Lower escape temperature (testing) ~ Lower escape temperature (ramping) x H_2S level

```
##
## Call:
## lm(formula = LET_Test ~ LET_Ramping * Treat_level, data = dat_period)
##
## Residuals:
##     Min      1Q  Median      3Q     Max
## -8.3965 -2.4839  0.7926  2.1082  6.4492
##
## Coefficients:
##                                   Estimate Std. Error t value Pr(>|t|)
## (Intercept)                         1.7671     0.7104   2.487 0.018096 *
## LET_Ramping                         0.7821     0.3389   2.308 0.027417 *
## Treat_levelH2S_0.02%               -3.8367     1.0481  -3.661 0.000872 ***
## LET_Ramping:Treat_levelH2S_0.02%  -0.3882     0.4985  -0.779 0.441683
## ---
## Signif. codes:  0 '***' 0.001 '**' 0.01 '*' 0.05 '.' 0.1 ' ' 1
```

```
##
## Residual standard error: 3.177 on 33 degrees of freedom
## Multiple R-squared:  0.3739, Adjusted R-squared:  0.317
## F-statistic:  6.57 on 3 and 33 DF,  p-value: 0.001321
```

2.4.4 Upper escape temperature (testing) ~ Upper escape temperature (ramping) x H_2S level

```
##
## Call:
## lm(formula = UET_Test ~ UET_Ramping * Treat_level, data = dat_period)
##
## Residuals:
##     Min      1Q  Median      3Q     Max
## -9.0588 -2.4412  0.4711  1.8661  6.6878
##
## Coefficients:
##                                Estimate Std. Error t value Pr(>|t|)
## (Intercept)                      1.9681     0.7999   2.460 0.019278 *
## UET_Ramping                      0.5993     0.3383   1.771 0.085721 .
## Treat_levelH2S_0.02%            -4.2691     1.1802  -3.617 0.000983 ***
## UET_Ramping:Treat_levelH2S_0.02%  -0.2044     0.5332  -0.383 0.703957
## ---
## Signif. codes:  0 '***' 0.001 '**' 0.01 '*' 0.05 '.' 0.1 ' ' 1
##
## Residual standard error: 3.576 on 33 degrees of freedom
## Multiple R-squared:  0.3369, Adjusted R-squared:  0.2766
## F-statistic: 5.589 on 3 and 33 DF,  p-value: 0.003261
```

2.4.5 Swim velocity (testing) ~ Swim velocity (ramping) x H_2S level

```
##
## Call:
## lm(formula = Velocity_Test ~ Velocity_Ramping * Treat_level,
##     data = dat_period)
##
## Residuals:
##      Min       1Q   Median       3Q      Max
## -0.31011 -0.08899 -0.01745  0.06981  0.67637
##
## Coefficients:
##                                     Estimate Std. Error t value Pr(>|t|)
## (Intercept)                         -0.04535    0.03929  -1.154   0.2567
## Velocity_Ramping                     0.13320    0.07422   1.795   0.0819 .
## Treat_levelH2S_0.02%                 0.10358    0.05797   1.787   0.0831 .
## Velocity_Ramping:Treat_levelH2S_0.02% -0.11541    0.10262  -1.125   0.2689
## ---
## Signif. codes:  0 '***' 0.001 '**' 0.01 '*' 0.05 '.' 0.1 ' ' 1
##
## Residual standard error: 0.1753 on 33 degrees of freedom
## Multiple R-squared:  0.1718, Adjusted R-squared:  0.09649
## F-statistic: 2.282 on 3 and 33 DF,  p-value: 0.0974
```

2.5 AQUATIC SURFACE RESPIRATION RATES BY TEMPERATURE AND H_2S LEVEL

2.5.1 Generalised linear model of ASR ~ Temperature x H_2S

```
##  Family: binomial
##   Links: mu = logit
## Formula: ASR | trials(n) ~ Temperature * H2S + (1 | ID/H2S/Temperature)
##    Data: dat_asr_summary (Number of observations: 26)
## Samples: 4 chains, each with iter = 10000; warmup = 5000; thin = 1;
##          total post-warmup samples = 20000
##
## Group-Level Effects:
## ~ID (Number of levels: 8)
##               Estimate Est.Error l-95% CI u-95% CI Rhat Bulk_ESS Tail_ESS
## sd(Intercept)     0.78      0.51     0.05     1.96 1.00     6594     7133
##
## ~ID:H2S (Number of levels: 14)
##               Estimate Est.Error l-95% CI u-95% CI Rhat Bulk_ESS Tail_ESS
## sd(Intercept)     0.66      0.41     0.04     1.60 1.00     4612     7210
##
## ~ID:H2S:Temperature (Number of levels: 26)
##               Estimate Est.Error l-95% CI u-95% CI Rhat Bulk_ESS Tail_ESS
## sd(Intercept)     0.97      0.24     0.60     1.54 1.00     6693    11074
##
## Population-Level Effects:
##                       Estimate Est.Error l-95% CI u-95% CI Rhat Bulk_ESS Tail_ESS
## Intercept                -4.23      0.64    -5.52    -2.98 1.00    11214    12595
## Temperature28             1.23      0.59     0.11     2.46 1.00     8877     8855
## H2S0.02                   2.85      0.75     1.40     4.40 1.00    10239    10855
## Temperature28:H2S0.02    -1.22      0.83    -2.96     0.38 1.00     8638     8930
##
## Samples were drawn using sampling(NUTS). For each parameter, Bulk_ESS
## and Tail_ESS are effective sample size measures, and Rhat is the potential
## scale reduction factor on split chains (at convergence, Rhat = 1).
```

BRMS fits logs odds ratio, so p=0.5 is a log odds ratio of 0.

The fixed effects odds ratios:

```
##                          Estimate Est.Error       Q2.5       Q97.5
## Intercept              0.01458133  1.889997 0.00402063  0.05062977
## Temperature28          3.43338547  1.812149 1.11365583 11.67793328
## H2S0.02               17.29011738  2.114111 4.06410976 81.21882613
## Temperature28:H2S0.02  0.29632204  2.303695 0.05159188  1.46613711
```

2.5.2 Fish temperature ~ log Shuttle rate x H_2S level

```
##
## Call:
## lm(formula = Temp.fish_Test ~ log_Shuttle_rt_Test * Treat_level,
##     data = dat_period)
##
```

```
## Residuals:
##     Min      1Q  Median      3Q     Max
## -6.1966 -2.1548 -0.1841  2.0549  8.0796
##
## Coefficients:
##                                          Estimate Std. Error t value Pr(>|t|)
## (Intercept)                                2.7898     0.8128   3.432  0.00163 **
## log_Shuttle_rt_Test                        0.8533     1.5117   0.564  0.57627
## Treat_levelH2S_0.02%                      -6.0573     1.2029  -5.036 1.66e-05 ***
## log_Shuttle_rt_Test:Treat_levelH2S_0.02%  -0.3261     2.9631  -0.110  0.91304
## ---
## Signif. codes:  0 '***' 0.001 '**' 0.01 '*' 0.05 '.' 0.1 ' ' 1
##
## Residual standard error: 3.626 on 33 degrees of freedom
## Multiple R-squared:  0.4362, Adjusted R-squared:  0.385
## F-statistic: 8.512 on 3 and 33 DF,  p-value: 0.0002506
```

2.5.3 Fish temperature ~ Swim velocity x H_2S level

```
##
## Call:
## lm(formula = Temp.fish_Test ~ Velocity_Test * Treat_level, data = dat_period)
##
## Residuals:
##     Min      1Q  Median      3Q     Max
## -6.5719 -2.4624  0.3197  1.8049  7.3732
##
## Coefficients:
##                                    Estimate Std. Error t value Pr(>|t|)
## (Intercept)                          2.7062     0.8377   3.231   0.0028 **
## Velocity_Test                       -1.0244     3.9146  -0.262   0.7952
## Treat_levelH2S_0.02%                -5.9709     1.2911  -4.625 5.55e-05 ***
## Velocity_Test:Treat_levelH2S_0.02%   1.3743     8.2767   0.166   0.8691
## ---
## Signif. codes:  0 '***' 0.001 '**' 0.01 '*' 0.05 '.' 0.1 ' ' 1
##
## Residual standard error: 3.642 on 33 degrees of freedom
## Multiple R-squared:  0.4313, Adjusted R-squared:  0.3796
## F-statistic: 8.342 on 3 and 33 DF,  p-value: 0.0002881
```

2.5.4 Fish temperature ~ Time ratio x H_2S level

```
##
## Call:
## lm(formula = Temp.fish_Test ~ Time_ratio_Test, data = dat_period)
##
## Residuals:
##     Min      1Q  Median      3Q     Max
## -6.9229 -1.5536 -0.1451  2.0979  4.1605
##
## Coefficients:
##                   Estimate Std. Error t value Pr(>|t|)
## (Intercept)     -1.851e-15  4.599e-01   0.000        1
```

```
## Time_ratio_Test  1.088e+01  1.367e+00   7.959 2.31e-09 ***
## ---
## Signif. codes:  0 '***' 0.001 '**' 0.01 '*' 0.05 '.' 0.1 ' ' 1
##
## Residual standard error: 2.797 on 35 degrees of freedom
## Multiple R-squared:  0.6441, Adjusted R-squared:  0.634
## F-statistic: 63.35 on 1 and 35 DF,  p-value: 2.311e-09
```

3 REANALYSIS OF H_2S (Short time series)

Some fish were removed prematurely during the H_2S experiments due to unusual behaviours. We reexamine the main responses by shortening the data set to a two-hour time window. This matches the ramping period time window and reduces the disparity in averaging period. We do not find qualitative differences if the response period is shortened in this way.

3.1 AVERAGE TREATMENT EFFECTS

3.1.1 Fish temperature ~ H_2S

```
##
## Call:
## lm(formula = Temp.fish ~ Treat_level, data = dat_CDD_short_testphase)
##
## Residuals:
##     Min      1Q  Median      3Q     Max
## -6.9751 -2.0715 -0.1875  1.7523  6.9571
##
## Coefficients:
##                      Estimate Std. Error t value Pr(>|t|)
## (Intercept)           27.5831     0.7333  37.613  < 2e-16 ***
## Treat_levelH2S_0.02%  -5.2421     1.0819  -4.845 2.56e-05 ***
## ---
## Signif. codes:  0 '***' 0.001 '**' 0.01 '*' 0.05 '.' 0.1 ' ' 1
##
## Residual standard error: 3.28 on 35 degrees of freedom
## Multiple R-squared:  0.4015, Adjusted R-squared:  0.3844
## F-statistic: 23.48 on 1 and 35 DF,  p-value: 2.558e-05
```

Confidence intervals:

```
##                          2.5 %    97.5 %
## (Intercept)           26.09432 29.071876
## Treat_levelH2S_0.02% -7.43849 -3.045743
```

3.1.2 Lower escape temperature ~ H_2S

```
##
## Call:
## lm(formula = LET ~ Treat_level, data = dat_CDD_short_testphase)
##
## Residuals:
##     Min      1Q  Median      3Q     Max
## -7.3020 -2.0668  0.6117  1.9390  5.9200
##
## Coefficients:
##                      Estimate Std. Error t value Pr(>|t|)
## (Intercept)            25.959      0.724  35.856  < 2e-16 ***
## Treat_levelH2S_0.02%   -3.581      1.068  -3.353  0.00193 **
## ---
## Signif. codes:  0 '***' 0.001 '**' 0.01 '*' 0.05 '.' 0.1 ' ' 1
```

```
##
## Residual standard error: 3.238 on 35 degrees of freedom
## Multiple R-squared:  0.2431, Adjusted R-squared:  0.2215
## F-statistic: 11.24 on 1 and 35 DF,  p-value: 0.001932
```

Confidence intervals:

```
##                          2.5 %    97.5 %
## (Intercept)          24.489424 27.428914
## Treat_levelH2S_0.02% -5.749174 -1.412582
```

3.1.3 Upper escape temperature ~ H_2S

```
##
## Call:
## lm(formula = UET ~ Treat_level, data = dat_CDD_short_testphase)
##
## Residuals:
##     Min      1Q  Median      3Q     Max
## -8.4519 -2.2231  0.5966  2.0844  6.1887
##
## Coefficients:
##                      Estimate Std. Error t value Pr(>|t|)
## (Intercept)            29.423      0.794  37.055  < 2e-16 ***
## Treat_levelH2S_0.02%   -4.062      1.171  -3.468  0.00141 **
## ---
## Signif. codes:  0 '***' 0.001 '**' 0.01 '*' 0.05 '.' 0.1 ' ' 1
##
## Residual standard error: 3.551 on 35 degrees of freedom
## Multiple R-squared:  0.2557, Adjusted R-squared:  0.2344
## F-statistic: 12.02 on 1 and 35 DF,  p-value: 0.00141
```

Confidence intervals:

```
##                          2.5 %    97.5 %
## (Intercept)          27.811329 31.035298
## Treat_levelH2S_0.02% -6.440164 -1.683885
```

3.1.4 log Shuttle rate ~ H_2S

```
##
## Call:
## lm(formula = log_Shuttle_rt ~ Treat_level, data = dat_CDD_short_testphase)
##
## Residuals:
##      Min       1Q   Median       3Q      Max
## -0.73323 -0.40682  0.09332  0.33121  0.77782
##
## Coefficients:
##                      Estimate Std. Error t value Pr(>|t|)
## (Intercept)            1.6363     0.1015  16.122   <2e-16 ***
## Treat_levelH2S_0.02%   0.1597     0.1497   1.066    0.294
```

```
## ---
## Signif. codes:  0 '***' 0.001 '**' 0.01 '*' 0.05 '.' 0.1 ' ' 1
##
## Residual standard error: 0.4539 on 35 degrees of freedom
## Multiple R-squared:  0.03147,    Adjusted R-squared:  0.003793
## F-statistic: 1.137 on 1 and 35 DF,  p-value: 0.2936
```

Confidence intervals:

```
##                            2.5 %    97.5 %
## (Intercept)           1.4302661 1.8423648
## Treat_levelH2S_0.02% -0.1443134 0.4636504
```

3.1.5 log Swim velocity ~ H_2S

```
##
## Call:
## lm(formula = log(Velocity) ~ Treat_level, data = dat_CDD_short_testphase)
##
## Residuals:
##      Min       1Q   Median       3Q      Max
## -0.73251 -0.34813 -0.03936  0.36610  1.57008
##
## Coefficients:
##                      Estimate Std. Error t value Pr(>|t|)
## (Intercept)           -1.5701     0.1315 -11.941 6.73e-14 ***
## Treat_levelH2S_0.02%   0.7142     0.1940   3.682 0.000775 ***
## ---
## Signif. codes:  0 '***' 0.001 '**' 0.01 '*' 0.05 '.' 0.1 ' ' 1
##
## Residual standard error: 0.588 on 35 degrees of freedom
## Multiple R-squared:  0.2792, Adjusted R-squared:  0.2586
## F-statistic: 13.56 on 1 and 35 DF,  p-value: 0.0007748
```

Confidence intervals (on metric scale):

```
##     2.5 %    97.5 %
## 0.2194626 0.8227460
```

3.1.6 Time ratio ~ H_2S

```
##
## Call:
## lm(formula = Time_ratio ~ Treat_level - 1, data = dat_CDD_short_testphase)
##
## Residuals:
##      Min       1Q   Median       3Q      Max
## -0.64245 -0.10876 -0.01446  0.13688  0.73988
##
## Coefficients:
##                      Estimate Std. Error t value Pr(>|t|)
## Treat_levelCTL_0%     0.07320    0.05582   1.311 0.198271
```

```
## Treat_levelH2S_0.02% -0.25812    0.06055  -4.263 0.000145 ***
## ---
## Signif. codes:  0 '***' 0.001 '**' 0.01 '*' 0.05 '.' 0.1 ' ' 1
##
## Residual standard error: 0.2496 on 35 degrees of freedom
## Multiple R-squared:  0.3624, Adjusted R-squared:  0.326
## F-statistic: 9.946 on 2 and 35 DF,  p-value: 0.0003799
```

Confidence intervals:

```
##                            2.5 %     97.5 %
## Treat_levelCTL_0%    -0.04012218  0.1865320
## Treat_levelH2S_0.02% -0.38103728 -0.1351963
```

3.2 REPEATABILITY (0% H_2S)

3.2.1 Fish temperature ramping->testing repeatability

```
##
## Repeatability estimation using the lmm method
##
## Call = rptR::rptGaussian(formula = Temp.fish ~ (1 | fishID), grname = "fishID", data = dat_short_icc
##
## Data: 40 observations
## ----------------------------------------
##
## fishID (20 groups)
##
## Repeatability estimation overview:
##       R     SE   2.5%  97.5% P_permut  LRT_P
##   0.651  0.139   0.32  0.836       NA  0.001
##
## Bootstrapping and Permutation test:
##             N   Mean Median   2.5%  97.5%
## boot     1000  0.631   0.65   0.32  0.836
## permut      1     NA     NA     NA     NA
##
## Likelihood ratio test:
## logLik full model = -92.24945
## logLik red. model = -97.43074
## D  = 10.4, df = 1, P = 0.000643
##
## ----------------------------------------
```

3.2.2 Fish log shuttle rate ramping->testing repeatability

```
##
## Repeatability estimation using the lmm method
##
## Call = rptR::rptGaussian(formula = log_Shuttle_rt ~ (1 | fishID), grname = "fishID", data = dat_shor
##
## Data: 40 observations
```

```
## ---------------------------------------
##
## fishID (20 groups)
##
## Repeatability estimation overview:
##       R     SE   2.5%  97.5% P_permut  LRT_P
##   0.526  0.163  0.138   0.77       NA  0.007
##
## Bootstrapping and Permutation test:
##             N   Mean Median   2.5%  97.5%
## boot     1000  0.503  0.517  0.138   0.77
## permut      1     NA     NA     NA     NA
##
## Likelihood ratio test:
## logLik full model = -31.01693
## logLik red. model = -33.99683
## D  = 5.96, df = 1, P = 0.00732
##
## ---------------------------------------
```

3.3 RESPONSES IN RAMPING AND TESTING PHASES

3.3.1 Fish temperature (testing) ~ Fish temperature (ramping) x H_2S level

```
##
## Call:
## lm(formula = Temp.fish_Test ~ Temp.fish_Ramping * Treat_level,
##     data = dat_period_short)
##
## Residuals:
##     Min      1Q  Median      3Q     Max
## -5.2895 -1.7023 -0.6633  1.7529  6.3004
##
## Coefficients:
##                                        Estimate Std. Error t value Pr(>|t|)
## (Intercept)                              2.5069     0.6253   4.009 0.000328 ***
## Temp.fish_Ramping                        0.9832     0.2805   3.505 0.001337 **
## Treat_levelH2S_0.02%                    -5.4019     0.9226  -5.855 1.48e-06 ***
## Temp.fish_Ramping:Treat_levelH2S_0.02%  -0.4618     0.4131  -1.118 0.271694
## ---
## Signif. codes:  0 '***' 0.001 '**' 0.01 '*' 0.05 '.' 0.1 ' ' 1
##
## Residual standard error: 2.793 on 33 degrees of freedom
## Multiple R-squared:  0.5906, Adjusted R-squared:  0.5534
## F-statistic: 15.87 on 3 and 33 DF,  p-value: 1.464e-06
```

3.3.2 log Shuttle rate (testing) ~ log Shuttle rate (ramping) x H_2S level

```
##
## Call:
## lm(formula = log_Shuttle_rt_Test ~ log_Shuttle_rt_Ramping * Treat_level,
##     data = dat_period_short)
##
```

```
## Residuals:
##      Min       1Q   Median       3Q      Max
## -0.65800 -0.29637  0.05306  0.28780  0.49492
##
## Coefficients:
##                                            Estimate Std. Error t value Pr(>|t|)
## (Intercept)                                -0.01442    0.07793  -0.185    0.854
## log_Shuttle_rt_Ramping                      0.66378    0.13909   4.772  3.6e-05 ***
## Treat_levelH2S_0.02%                        0.05273    0.11615   0.454    0.653
## log_Shuttle_rt_Ramping:Treat_levelH2S_0.02% -0.20421    0.24621  -0.829    0.413
## ---
## Signif. codes:  0 '***' 0.001 '**' 0.01 '*' 0.05 '.' 0.1 ' ' 1
##
## Residual standard error: 0.3441 on 33 degrees of freedom
## Multiple R-squared:  0.4751, Adjusted R-squared:  0.4274
## F-statistic: 9.957 on 3 and 33 DF,  p-value: 8.008e-05
```

3.3.3 Lower escape temperature (testing) ~ Lower escape temperature (ramping) x H_2S level

```
##
## Call:
## lm(formula = LET_Test ~ LET_Ramping * Treat_level, data = dat_period_short)
##
## Residuals:
##     Min      1Q  Median      3Q     Max
## -6.1463 -1.3860  0.2559  1.7706  6.2120
##
## Coefficients:
##                                 Estimate Std. Error t value Pr(>|t|)
## (Intercept)                       1.6656     0.6364   2.617 0.013283 *
## LET_Ramping                       0.9867     0.3036   3.250 0.002655 **
## Treat_levelH2S_0.02%             -3.6116     0.9389  -3.846 0.000519 ***
## LET_Ramping:Treat_levelH2S_0.02%  -0.5559     0.4466  -1.245 0.221986
## ---
## Signif. codes:  0 '***' 0.001 '**' 0.01 '*' 0.05 '.' 0.1 ' ' 1
##
## Residual standard error: 2.846 on 33 degrees of freedom
## Multiple R-squared:  0.4485, Adjusted R-squared:  0.3984
## F-statistic: 8.947 on 3 and 33 DF,  p-value: 0.0001763
```

3.3.4 Upper escape temperature (testing) ~ Upper escape temperature (ramping) x H_2S level

```
##
## Call:
## lm(formula = UET_Test ~ UET_Ramping * Treat_level, data = dat_period_short)
##
## Residuals:
##     Min      1Q  Median      3Q     Max
## -7.1390 -1.6313 -0.1459  1.8910  7.3691
##
## Coefficients:
##                                 Estimate Std. Error t value Pr(>|t|)
## (Intercept)                       1.9104     0.7444   2.567 0.014995 *
```

```
## UET_Ramping                         0.7389     0.3148   2.347 0.025075 *
## Treat_levelH2S_0.02%               -4.1374     1.0983  -3.767 0.000648 ***
## UET_Ramping:Treat_levelH2S_0.02%  -0.2942     0.4962  -0.593 0.557313
## ---
## Signif. codes:  0 '***' 0.001 '**' 0.01 '*' 0.05 '.' 0.1 ' ' 1
##
## Residual standard error: 3.328 on 33 degrees of freedom
## Multiple R-squared:  0.3837, Adjusted R-squared:  0.3277
## F-statistic: 6.848 on 3 and 33 DF,  p-value: 0.001031
```

3.3.5 Swim velocity (testing) ~ Swim velocity (ramping) x H_2S level

```
##
## Call:
## lm(formula = Velocity_Test ~ Velocity_Ramping * Treat_level,
##     data = dat_period_short)
##
## Residuals:
##      Min       1Q   Median       3Q      Max
## -0.37951 -0.11081 -0.03749  0.05146  0.68991
##
## Coefficients:
##                                        Estimate Std. Error t value Pr(>|t|)
## (Intercept)                           -0.074704   0.043118  -1.733   0.0925 .
## Velocity_Ramping                       0.121011   0.081458   1.486   0.1469
## Treat_levelH2S_0.02%                   0.162390   0.063618   2.553   0.0155 *
## Velocity_Ramping:Treat_levelH2S_0.02%  0.004758   0.112630   0.042   0.9666
## ---
## Signif. codes:  0 '***' 0.001 '**' 0.01 '*' 0.05 '.' 0.1 ' ' 1
##
## Residual standard error: 0.1924 on 33 degrees of freedom
## Multiple R-squared:  0.2694, Adjusted R-squared:  0.203
## F-statistic: 4.057 on 3 and 33 DF,  p-value: 0.01468
```

4 ANALYSIS OF HYPOXIA (2% O_2)

Shuttlebox data collected by Joshua C. Shaw

Analysis performed by Dimitri A. Skandalis

4.1 AVERAGE TREATMENT EFFECTS

4.1.1 Fish temperature ~ O_2

```
##
##  Welch Two Sample t-test
##
## data:  Temp.fish by Treat_level
## t = 2.0056, df = 11.384, p-value = 0.03463
## alternative hypothesis: true difference in means is greater than 0
## 95 percent confidence interval:
##  0.3388087       Inf
## sample estimates:
## mean in group NOX_21%  mean in group HPX_2%
##            -0.4819944            -3.6385575
```

4.1.2 Lower escape temperature ~ O_2

```
##
##  Welch Two Sample t-test
##
## data:  LET by Treat_level
## t = 2.0246, df = 13.882, p-value = 0.03129
## alternative hypothesis: true difference in means is greater than 0
## 95 percent confidence interval:
##  0.2355009       Inf
## sample estimates:
## mean in group NOX_21%  mean in group HPX_2%
##             -1.787126             -3.605332
```

4.1.3 Upper escape temperature ~ O_2

```
##
##  Welch Two Sample t-test
##
## data:  UET by Treat_level
## t = 1.8713, df = 13.802, p-value = 0.04133
## alternative hypothesis: true difference in means is greater than 0
## 95 percent confidence interval:
##  0.09558922        Inf
## sample estimates:
## mean in group NOX_21%  mean in group HPX_2%
##             1.2600558            -0.3938188
```

4.1.4 Swim velocity ~ O_2

```
##
##  Welch Two Sample t-test
##
## data:  Velocity by Treat_level
## t = 1.8521, df = 13.895, p-value = 0.04269
## alternative hypothesis: true difference in means is greater than 0
## 95 percent confidence interval:
##  0.04384515        Inf
## sample estimates:
## mean in group NOX_21%  mean in group HPX_2%
##            -0.4857143            -1.3900000
```

4.1.5 log Shuttle rate ~ O_2

```
##
##  Welch Two Sample t-test
##
## data:  log_Shuttle_rt by Treat_level
## t = 1.543, df = 14.585, p-value = 0.07212
## alternative hypothesis: true difference in means is greater than 0
## 95 percent confidence interval:
##  -0.03660821         Inf
## sample estimates:
## mean in group NOX_21%  mean in group HPX_2%
##           -0.03416448           -0.29898407
```

4.1.6 Side preference ~ O_2

```
##
##  Welch Two Sample t-test
##
## data:  Time_ratio by Treat_level
## t = 1.091, df = 9.529, p-value = 0.1511
## alternative hypothesis: true difference in means is greater than 0
## 95 percent confidence interval:
##  -0.06543593         Inf
## sample estimates:
## mean in group NOX_21%  mean in group HPX_2%
##            0.03609128           -0.06161372
```
