# Supplementary figures and images for: Hydrogen sulfide exposure reduces thermal set point in zebrafish
Source: R Soc Open Sci. 2020 Nov 4;7(11):200416. doi: 10.1098/rsos.200416 (PMC7735326; doi:10.1098/rsos.200416)

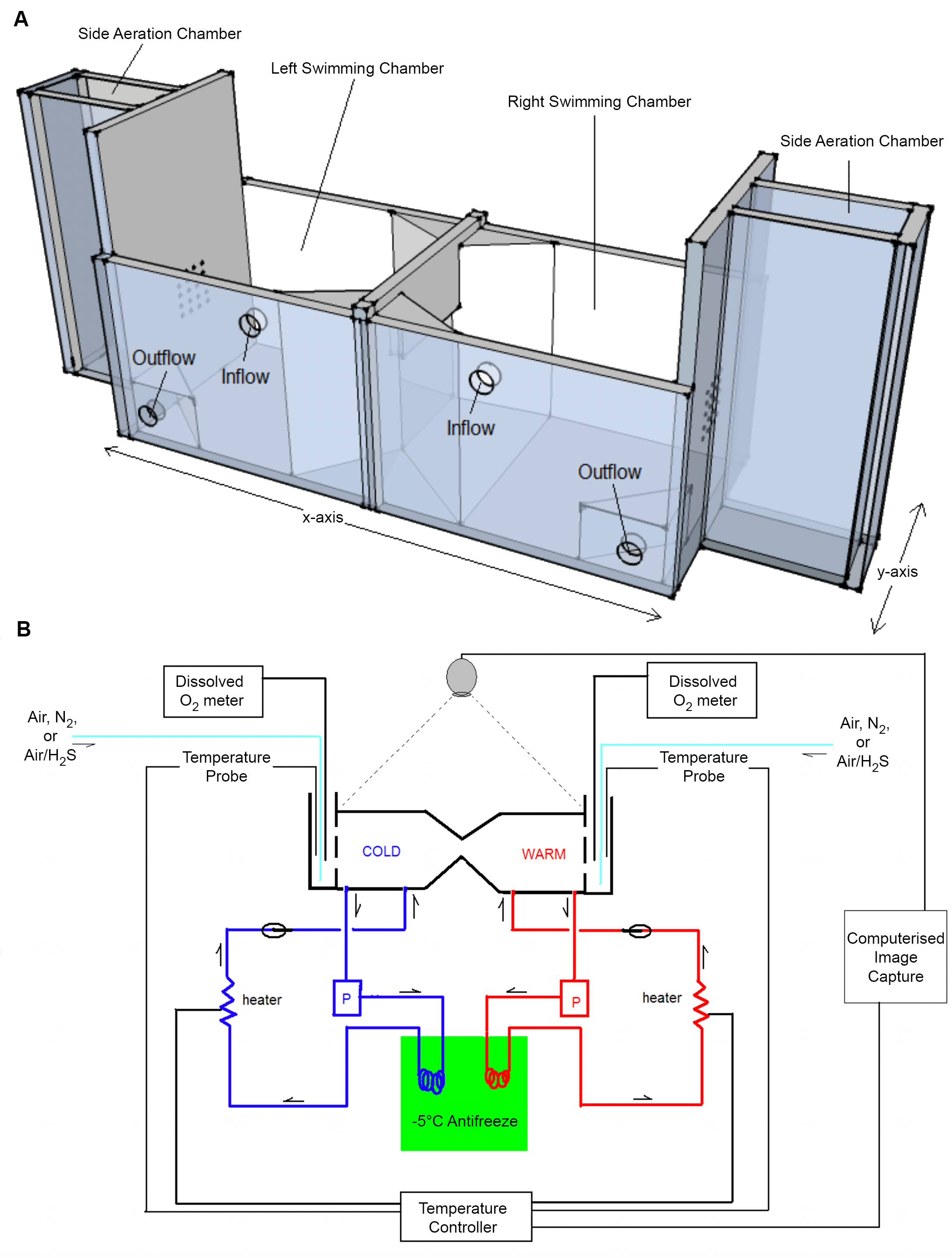

Supplement: Figure S1 [file rsos200416supp2.png]

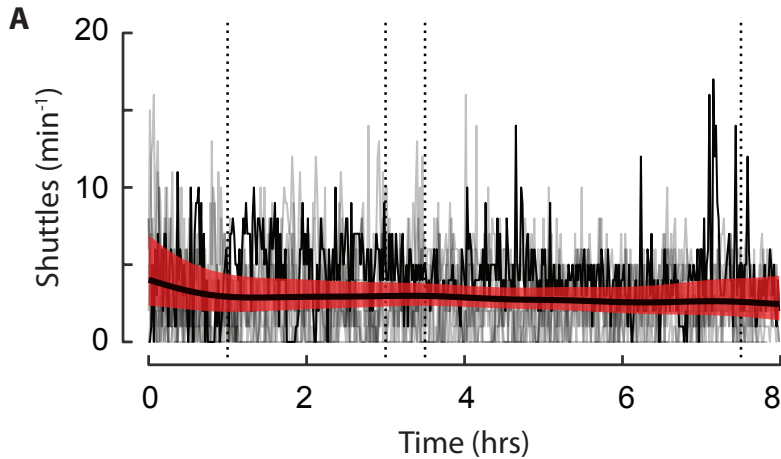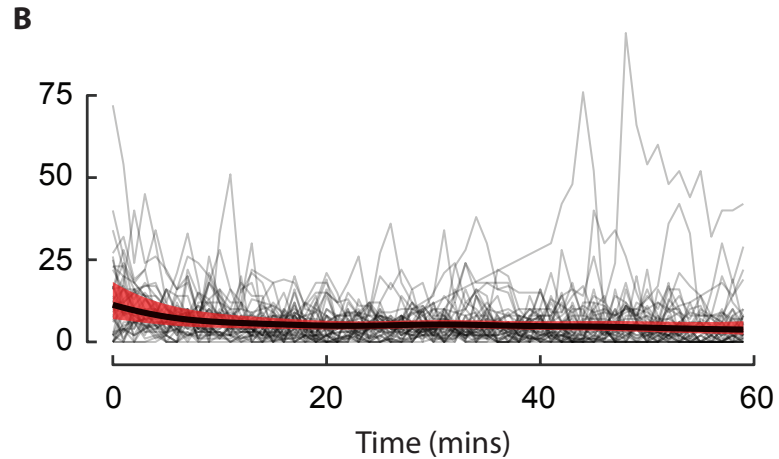

Supplement: Figure S2 [file rsos200416supp3.pdf]
